# Single-cell profiling of human gliomas reveals macrophage ontogeny as a basis for regional differences in macrophage activation in the tumor microenvironment

**DOI:** 10.1186/s13059-017-1362-4

**Published:** 2017-12-20

**Authors:** Sören Müller, Gary Kohanbash, S. John Liu, Beatriz Alvarado, Diego Carrera, Aparna Bhaduri, Payal B. Watchmaker, Garima Yagnik, Elizabeth Di Lullo, Martina Malatesta, Nduka M. Amankulor, Arnold R. Kriegstein, Daniel A. Lim, Manish Aghi, Hideho Okada, Aaron Diaz

**Affiliations:** 1 Department of Neurological Surgery, University of California, San Francisco, CA 94143 USA; 2 Eli and Edythe Broad Center of Regeneration Medicine and Stem Cell Research, University of California, San Francisco, CA 94158 USA; 3 Department of Neurology, University of California, San Francisco, CA 94158 USA; 4 University of California, San Francisco, CA 94158 USA; 5 Veterans Affairs Medical Center, San Francisco, CA 94121 USA; 6 Department of Neurological Surgery, University of Pittsburgh, Pittsburgh, PA 15261 USA

**Keywords:** Glioma, Single-cell sequencing, Immunotherapy, Macrophage

## Abstract

**Background:**

Tumor-associated macrophages (TAMs) are abundant in gliomas and immunosuppressive TAMs are a barrier to emerging immunotherapies. It is unknown to what extent macrophages derived from peripheral blood adopt the phenotype of brain-resident microglia in pre-treatment gliomas. The relative proportions of blood-derived macrophages and microglia have been poorly quantified in clinical samples due to a paucity of markers that distinguish these cell types in malignant tissue.

**Results:**

We perform single-cell RNA-sequencing of human gliomas and identify phenotypic differences in TAMs of distinct lineages. We isolate TAMs from patient biopsies and compare them with macrophages from non-malignant human tissue, glioma atlases, and murine glioma models. We present a novel signature that distinguishes TAMs by ontogeny in human gliomas. Blood-derived TAMs upregulate immunosuppressive cytokines and show an altered metabolism compared to microglial TAMs. They are also enriched in perivascular and necrotic regions. The gene signature of blood-derived TAMs, but not microglial TAMs, correlates with significantly inferior survival in low-grade glioma. Surprisingly, TAMs frequently co-express canonical pro-inflammatory (M1) and alternatively activated (M2) genes in individual cells.

**Conclusions:**

We conclude that blood-derived TAMs significantly infiltrate pre-treatment gliomas, to a degree that varies by glioma subtype and tumor compartment. Blood-derived TAMs do not universally conform to the phenotype of microglia, but preferentially express immunosuppressive cytokines and show an altered metabolism. Our results argue against status quo therapeutic strategies that target TAMs indiscriminately and in favor of strategies that specifically target immunosuppressive blood-derived TAMs.

**Electronic supplementary material:**

The online version of this article (doi:10.1186/s13059-017-1362-4) contains supplementary material, which is available to authorized users.

## Background

The cellular heterogeneity of tumor-associated macrophages (TAMs) is a critical roadblock to the development of cancer immunotherapies. For example, macrophage colony-stimulating factor, a hematopoietic growth factor that promotes macrophage survival, is over-expressed in glioma. Murine gliomas can be regressed by inhibiting colony-stimulating factor receptor (CSF1R) [1]. However, clinical trials targeting CSF1R have so far failed to increase overall survival [2]. Evidence suggests that subpopulations of TAMs are resistant to CSF1R inhibition [3]. Another example is acquired resistance to the anti-angiogenesis therapy bevacizumab. Here, blood-derived TAMs preferentially contribute to therapy resistance, relative to brain-resident microglia [4]. Thus, TAM heterogeneity is a barrier to effective immunotherapies. Moreover, CSF1R blockade exemplifies the status quo, which seeks to target TAMs indiscriminately even though TAMs can play both tumor-supportive and anti-tumor roles.

In glioma, TAMs comprise two populations: brain-resident microglia, whose progenitors migrate to the central nervous system (CNS) during early development [5]; and macrophages that differentiate from bone marrow-derived monocytes, that have extravasated the blood–brain barrier [6]. Studies of the differences between these two populations have been confounded by a lack of specific markers to separately purify these cell types from human gliomas [7]. How ontogeny contributes to TAM education in the glioma microenvironment is not fully understood.

There have been mixed reports of the degree to which bone marrow-derived macrophages (BMDM) contribute to the TAM pool in murine gliomas. Irradiation followed by a transfer of labeled bone marrow was used to show that the majority of TAMs are brain-resident microglia [8]. On the other hand, macrophage lineage-tracing, using a genetic system that does not involve irradiation, demonstrated that BMDM do infiltrate murine gliomas to a significant extent [9]. It is unclear to what extent BMDMs infiltrate untreated human gliomas. It is unknown whether BMDMs adopt the phenotype of brain-resident microglia in malignant conditions.

To address this, we applied single-cell RNA-sequencing (scRNA-seq) to pre-treatment human gliomas. We compared gene expression in TAMs to microglia and macrophages, derived from non-malignant human tissue. We integrated our scRNA-seq with published glioma cohorts and lineage tracing studies in mouse. We correlated TAM composition with glioma molecular subtype and overall survival. Using public glioma atlases, we mapped TAM signatures to tumor anatomical structures and identified recurrent regional variation in TAM composition.

We found that human TAMs in vivo exhibit both canonical and non-canonical activation states, yet express durable markers of lineage. We present novel gene signatures that are specific to human TAMs of bone marrow and microglial origin, respectively. Blood-derived TAMs significantly infiltrate pre-treatment gliomas. Their infiltration correlates with tumor grade and varies by glioma subtype. Compared to TAMs of microglial origin, blood-derived TAMs upregulate immunosuppressive cytokines and markers of an oxidative metabolism, characteristic of the M2 phenotype. Blood-derived TAMs aggregate in perivascular and necrotic regions, compared to microglia. Elevated expression of blood-derived-TAM markers, but not microglial-TAM marker genes, correlates with significantly inferior overall survival in grade II–III low-grade glioma (LGG). Taken together, these results support targeting immunosuppressive TAMs derived from peripheral blood and therapies that normalize the blood–brain barrier.

## Results

### Single-cell sequencing produces a transcriptome-wide assessment of TAM expression patterns in vivo

We endeavored to assess both inter- and intra-tumor TAM heterogeneity, by assembling a cohort that spanned glioma grades and molecular subtypes. We profiled TAMs from patient biopsies, derived from 13 untreated primary gliomas (11 glioblastomas [GBMs], two LGGs; Additional file 1: Table S1), and combined this with public data from an additional 580 glioma cases. We performed scRNA-seq on seven of the in-house cases (five GBMs, two LGGs) [10, 11]. ScRNA-seq data were available from 12 published LGG cases [12, 13].

To robustly assess the in-house cases, we applied two orthogonal scRNA-seq platforms: the Fluidigm C1 (which produces full-transcript coverage); and the 10X Genomics platform (which produces 3’ tagged data, but at a higher cellular throughput). We performed C1-based scRNA-seq for three primary GBMs and one primary grade 3 oligodendroglioma. Additionally, we used 10X-based scRNA-seq to profile two primary GBMs and one primary grade 2 astrocytoma.

**Fig. 1:**
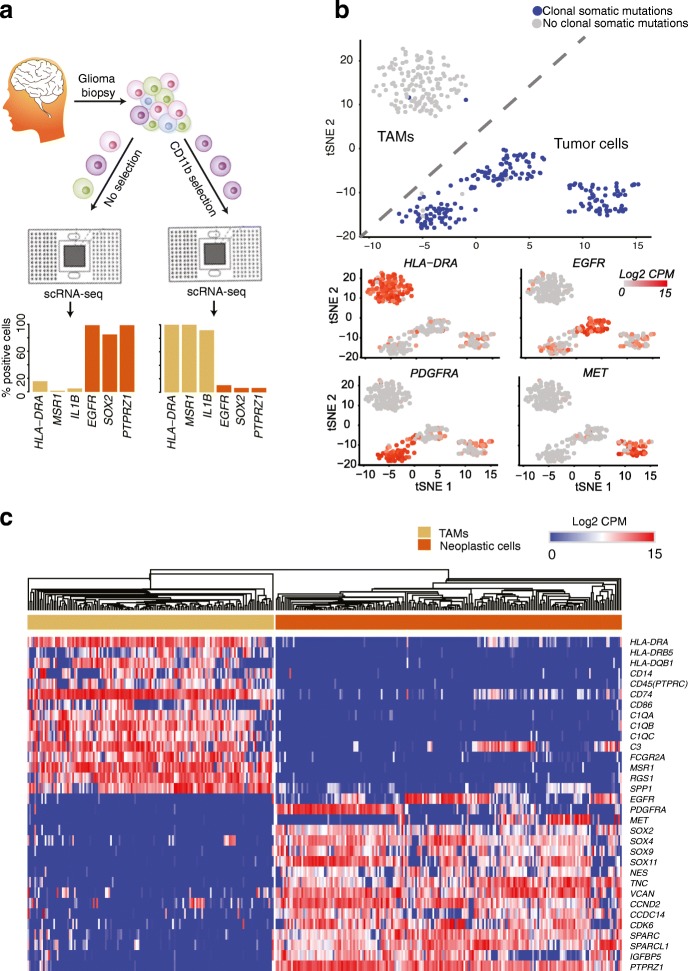
ScRNA-seq of neoplastic and immune cells from human primary gliomas. **a** Both whole-tumor and CD11b-purified single-cell suspensions, derived from glioma biopsies, were subjected to scRNA-seq (*top*) allowing for quantification of markers in single cells from both populations (*bottom*). **b** t-distributed stochastic neighbor embedding plot of cells from whole-tumor and CD11b-purified scRNA-seq, colored by the presence of somatic mutations that are clonal in exome sequencing (*top*) or by the expression of canonical marker genes (*bottom*), measured in counts per million (CPM). **c** Hierarchical clustering of cells (*columns*), grouped by their expression of canonical marker genes (*rows*)

To increase the number of TAMs profiled, we additionally purified TAMs from four of the cases (two GBMs, one G3 oligo., one G2 astro.), using the canonical macrophage marker CD11b (see “Methods” and Fig. 1a). We validated our isolation protocol via flow cytometry, and the purity of CD11b-expressing cells was over 96% (Additional file 2: Figure S1a). For these cases, we separately performed scRNA-seq on both a whole-tumor suspension and a CD11b-purified suspension.

We next sought to purify TAMs in silico from the whole-tumor scRNA-seq. Additionally, we wanted to filter any non-TAMs that were inadvertently sequenced in the CD11b-purified scRNA-seq. We took separate approaches for the C1 and 10X data.

We obtained 672 cells from the C1-based scRNA-seq. We removed 206 cells with low sequencing depth and/or low transcript diversity [14]. We then separated scRNA-seq libraries based on two techniques: (1) clustering by gene expression; and (2) analysis of clonal, somatic mutations identified in matched exome-sequencing (exome-seq) data (see “Methods”). To compare expressed mutations between exome-seq and scRNA-seq data, we used our previously described methodology [15, 16].

We found that putative TAMs identified from the whole-tumor scRNA-seq clustered together with TAMs sequenced from the CD11b + suspensions and away from putative neoplastic cells from the whole-tumor scRNA-seq. Both t-distributed stochastic neighbor embedding (tSNE) and hierarchical clustering in a space of canonical markers show a clear separation between neoplastic and TAM populations (Fig. 1b, c). Putative TAMs are devoid of expressed somatic mutations, but robustly express class II human-leukocyte antigen and other macrophage-specific genes. On the other hand, putative neoplastic cells express somatic mutations identified as clonal in exome-seq and express high levels of receptor-tyrosine kinases. For all downstream analysis of TAMs from the C1 platform, we exclusively used those cells (n = 142) that robustly expressed TAM markers and were devoid of somatic mutations.

For the 10X data, we initially filtered non-TAMs from the CD11b-purified scRNA-seq, based on the expression of canonical macrophage markers (Additional file 2: Figure S1b). In agreement with our CD11b-purity assessments via flow cytometry, 91% of cells (n = 907) were identified as TAMs. We then performed transcriptional clustering of the TAMs from the CD11b-purified scRNA-seq, together with cells from the whole-tumor 10X-based scRNA-seq. This identified an additional 3132 TAMs, which clustered together with TAMs from the CD11b-purified 10X-based scRNA-seq and robustly expressed canonical macrophage markers (Additional file 2: Figure S1c). To test for potential batch effects, we compared two independent 10x captures (Additional file 2: Figure S1d) from the same tumor sample (SF11136). The cells aggregated in run-independent clusters, pointing to limited technical variance introduced by single-cell capture and sequencing.

Lastly, we retrieved published data from scRNA-seq of TAMs from nine astrocytomas (*n* = 1039 cells) and three oligodendrogliomas (*n* = 235 cells) [12, 13]. The identity of these cells had been previously determined by Venteicher et al. based on an absence of somatic mutations, and the expression of macrophage markers [17], which we confirmed (Additional file 2: Figure S1e). The final scRNA-seq dataset used for all subsequent analysis comprises 5455 TAMs (1274 published cells and 4181 novel cells) from 19 patients.

### A gene signature that distinguishes TAMs by ontogeny in mice is conserved in human glioma

Lineage tracing and RNA sequencing (RNA-seq) has been used to isolate and profile BMDM and microglia from murine glioma models [9]. Bowman et al. used both irradiation-based and genetic lineage-tracing systems. They found 836 genes that were differentially expressed between BMDM and microglial TAMs, recurrently in both models (Additional file 3: Table S2). We reasoned that these genes would contain a core signature of lineage identity that might be conserved in human. We compared homologues of these murine TAM genes to genes expressed in human macrophages.

**Fig. 2:**
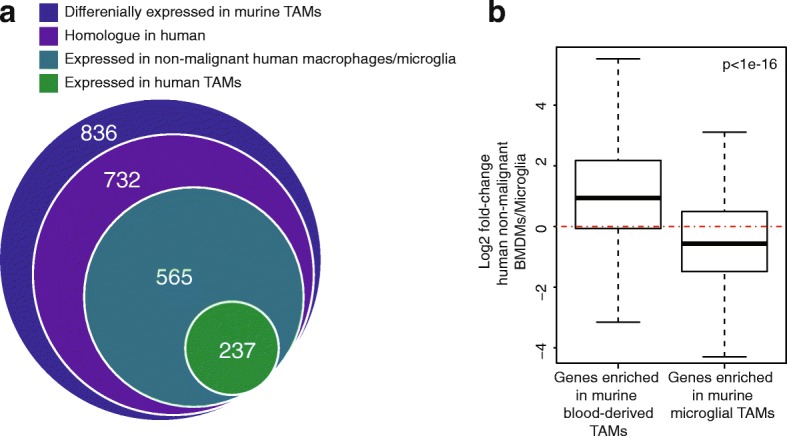
Analysis of published data identifies markers of ontogeny. **a** The intersection of: (1) genes that are differentially expressed between blood-derived and microglial TAMs in mouse (outer circle); (2) their homologues; (3) genes expressed (mean CPM > 1) in human BMDM/microglia from non-malignant tissue; and (4) TAMs from human gliomas (*n* = 16 patients). **b** Distributions of the log2 ratios (human BMDMs over microglia) (*y-axis*) for the differentially expressed murine-TAM homologues from (**a**)

We found that 237 of the lineage-specific murine-TAM genes had homologues that were expressed in human TAMs (Fig. 2a). On the other hand, 565 of the lineage-specific mouse genes were expressed by human macrophages of some ontogeny [18–21], in non-malignant conditions. On average, genes that are differentially expressed in mouse are also differentially expressed between human BMDM and microglia from non-malignant tissue (Fig. 2b). However, this agreement is not universally the case, underscoring the need to compare murine models with studies of human clinical samples.

To resolve whether the 237 mouse homologues are sufficient to identify discrete subpopulations of human TAMs, we performed principal component analysis (PCA) in the space of those genes using our scRNA-seq data (Additional file 2: Figure S2a). Gaussian mixture modeling of the resulting sample scores, along principal component 1 (PC1), showed two distinct subpopulations. To determine the utility of combining the C1 and Smart-seq2 datasets, we performed multiple factor analysis (MFA), a generalization of PCA used to combine multiple tables of measurements (Additional file 2: Figure S2b). We found that the per table contributions to variance explained from each of the datasets were approximately equal (ratio of partial inertias = 0.832).

**Fig. 3:**
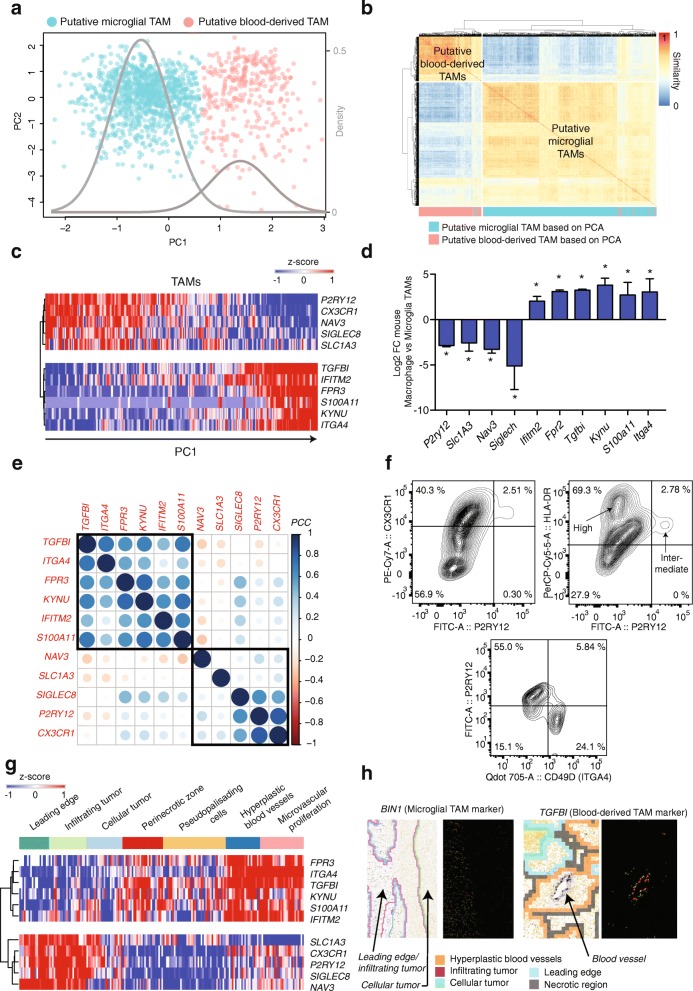
A gene signature to separate TAMs by ontogeny in mouse and human gliomas. **a** PCA of human TAMs in the space of genes that are ontogeny-specific in murine gliomas. The density curves of a Gaussian mixture model are in *gray*. **b** Consensus clustering of TAMs in the space of genes that are ontogeny-specific in murine gliomas. PCA-based cluster assignments from (**a**) are indicated by *color*. **c** *Heatmap* of the average expression (z-score) of indicated genes in windows of ten cells, sorted according to their PC1 score. **d** Log2 ratios of gene expression in murine blood-derived TAMs over murine microglial TAMs, averaged over the mouse models of Bowman et al. * = adjusted *p* value < 0.05 in both mouse models. *Error bars* indicate standard error of the mean. **e** Pearson correlation coefficients, computed via RNA-seq of LGGs and GBMs from TCGA (n = 558 cases). Genes are ordered by hierarchical clustering, *boxes* indicate a dendogram cut obtaining two clusters. **f** *Top left*: Flow cytometric analysis of TAMs gated on live CD11b + myeloid cells from a primary GBM (SF10941) stained for P2RY12 and CX3CR1. *Top right*: Flow cytometric analysis of TAMs gated on live CD11b + myeloid cells from a primary GBM (SF10941) stained for P2RY12 and HLA-DR. *Bottom*: Flow cytometric analysis of TAMs gated on live CD11b + myeloid cells from a primary GBM (SF11425) stained for P2RY12 and CD49D (encoded by *ITGA4*). **g** Gene expression from the Ivy Glioblastoma Atlas Project. Each column annotates expression in RNA‐seq of an anatomically defined tumor compartment. **h** In situ hybridization for *BIN1* and *TGFBI* in anatomically annotated regions (indicated by *color*) for two primary GBMs

In a PCA of the combined table, we found that PC1 stratified TAMs into two distinct platform-independent populations (Fig. 3a, Additional file 2: Figure S2a). The intersection of these two clusters, as estimated by a Gaussian mixture model, is < 5%. A consensus clustering of TAMs in the space of the 237 homologues recapitulates the clustering identified via PCA (Matthew’s correlation 0.946, Fig. 3b). Markers of murine microglial TAMs are enriched in genes that negatively load PC1, while makers of murine blood-derived TAMs are enriched in genes that positively load PC1 (Fisher’s exact test *p* < 1e-4). Thus, genes differentially expressed between murine TAM lineages also distinguish two populations in human gliomas.

We identified 66 genes that strongly loaded PC1 (Fig. 3c, Additional file 2: Figure S3c, Additional file 4: Table S3, Additional file 5: Table S4), which were differentially expressed between blood-derived and murine microglial TAMs (Fig. 3d), and which were tightly correlated across human gliomas in RNA-seq data from The Cancer Genome Atlas (TCGA) (Fig. 3e, Additional file 2: Figure S3a) [22]. A PCA of the 10X-derived scRNA-seq, also using the same set of 237 homologues, validated our expression signature. Here, too, PC1 identifies two populations, distinguished by the expression of our core signature genes (Additional file 2: Figure S3b). We propose these 66 genes (Additional file 5: Table S4) as core markers of lineage, as they are differentially expressed between microglial and blood-derived macrophages, in human and in mouse, in malignant and in non-malignant tissue.

CX3CR1 is widely used to isolate murine microglia in both non-malignant [23] and malignant conditions [24]. In human tissue, however, it is known that CX3CR1 is expressed by monocytes and its expression increases during differentiation into macrophages; thus, isolation of human microglial TAMs via CX3CR1 alone may represent an enrichment more than a purification [24–26]. On the other hand, P2RY12 came up in all of our analyses as a specific marker of microglial TAMs. Also, P2RY12 is known to be specific to microglia vs bone-marrow macrophages in non-malignant tissues [27, 28]. To determine if P2RY12 was expressed by human microglial TAMs at the protein level, we performed multicolor flow cytometry for CD11b, P2RY12, and CX3CR1 on leukocytes isolated from a human GBM-biopsy (SF10941). We found three distinct populations of TAMs (Fig. 3f, top left). One population of CD11b + cells is P2RY12- and CX3CR1- (putative CX3CR1- BMDM), one CD11b + population is CX3CR1+ and P2RY12- (putative CX3CR1+ BMDM), and one population is CD11b+/CX3CR1+/P2RY12+ (putative microglia).

We also stained for HLA-DRA in SF10941, a core component of class II human-leukocyte antigen, which our scRNA-seq data predicted was enriched in blood-derived TAMs relative to microglia. We found that P2RY12+ microglia express intermediate HLA-DR levels, while P2RY12– macrophages are characterized by high HLA-DR levels (Fig. 3f, top right). Additionally, we performed analytical flow cytometry on a GBM biopsy from an additional patient (SF11425), staining for CD11b, P2RY12, and the blood-derived macrophage marker CD49D (encoded by *ITGA4*). We found two main populations of CD11b + cells: P2RY12+ CD49D– cells and CD49D+ P2RY12– cells, underlining the ability of these two markers to distinguish macrophages and microglia on the protein level (Fig. 3f, bottom).

### TAMs of distinct ontogenies are enriched in distinct tumor-anatomical structures

We quantified our TAM-lineage signature in data from the Ivy Glioblastoma Atlas Project (IGAP) [29]. IGAP researchers have performed RNA-seq on microdissections of specific glioma anatomical structures, identified from hematoxylin and eosin (H&E) staining. Gene markers of human microglial TAMs are enriched in samples from the leading edge of invading gliomas and in adjacent infiltrated white matter. Genes specific to blood-derived TAMs are localized in regions of hyperplastic blood vessels, microvascular proliferation, and in peri-necrotic regions (Fig. 3g, Additional file 2: Figure S3d).

Additionally, we assessed in situ hybridizations for *TGFBI* and *BIN1* in glioma tissue sections from IGAP. These two genes are lineage markers for macrophages and microglia, respectively, from our 66-gene signature. As predicted, we found enrichment of *TFGBI* near putative blood vessels. Moreover, *BIN1* is enriched in infiltrated white matter and its expression decreases rapidly in the cellular tumor (Fig. 3h).

### TAMs of distinct ontogenies express distinct gene programs

**Fig. 4:**
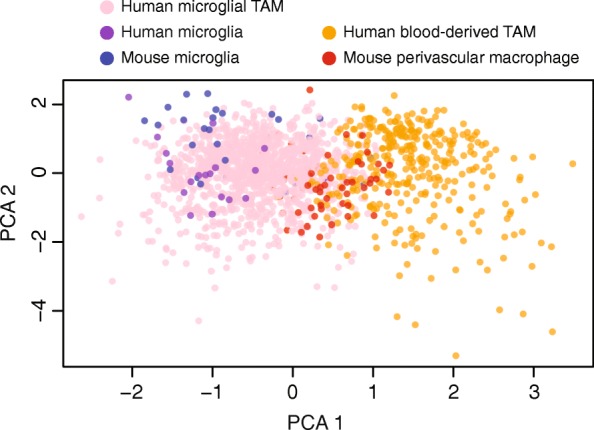
Markers of ontogeny from human TAMs also separate brain-derived perivascular macrophages from microglia in scRNA-seq of mouse and human non-malignant cortex. A PCA of human TAMs (*orange*/*pink*, *n* = 1416 cells), human microglia from non-malignant cortex (*purple*, *n* = 17 cells), murine microglia from non-malignant cortex (*blue*, *n* = 33 cells), and murine perivascular macrophages from non-malignant cortex (*red*, *n* = 65 cells). PCA was performed in the space of 87 genes that are differentially expressed between murine-TAM lineages and robustly measured across all datasets (mean CPM > 1 in all datasets)

We found that our lineage signature also separates brain-derived perivascular macrophages from microglia, in scRNA-seq data from mouse [30] and human [31] non-malignant cortex (Fig. 4). Like our blood-derived TAMs, these perivascular macrophages arose from peripheral monocytes that permeated the blood–brain barrier [30]. Consistent with this common lineage, both human blood-derived TAMs and murine perivascular macrophages express a common gene signature. In our data, both cell types upregulate the phagocytic receptor CD93, relative to microglia. This is in accord with the known role of perivascular macrophages as constitutive phagocytes [32, 33]. Likewise, mouse and human microglia from non-malignant brain share a signature of their lineage that is conserved in microglial TAMs (Additional file 6: Table S5), including *P2RY12*.

A differential-expression test between human blood-derived and microglial TAMs (DESeq adj. *p* value < 1e-3) confirmed a phagocytic phenotype in blood-derived TAMs. Blood-derived TAMs upregulate numerous structural components of the phagolysosome and a variety of phagocytosis-promoting receptors (Additional file 2: Figure S4a), compared to microglial TAMs. Intriguingly, blood-derived TAMs express significantly higher levels of genes typically associated with an immunosuppressive, alternatively activated (M2) phenotype, such as *IL10* and *TGFB2*, compared to microglia (Additional file 7: Table S6).

An activated tricarboxylic acid (TCA) cycle is a hallmark of M2 macrophage metabolism. Conversely, in classically activated (M1) macrophages, the TCA cycle is broken in two places: after citrate production and again after succinate production [34, 35]. Blood-derived TAMs show significantly elevated levels of genes that are rate-limiting for citrate and succinate processing at exactly these two breakpoints (Additional file 2: Figure S4b). This suggests an activation of the TCA cycle in in blood-derived TAMs.

### The gene signature for blood-derived TAMs varies by glioma subtype and correlates with significantly shorter overall survival in LGG

**Fig. 5:**
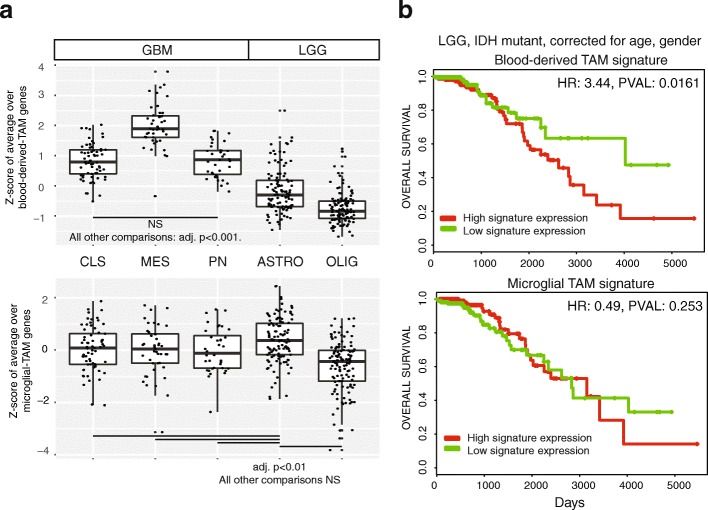
Infiltration of blood-derived TAMs varies by glioma molecular subtype and correlates with inferior survival. **a** Z-scores of averages over blood-derived (*top*) and microglial (*bottom*)-TAM signature genes, compared across glioma subtypes (*n* = 371 cases, 117 oligodendrogliomas [OLIGs], 110 astrocytomas [ASTROs], 144 GBMs). *CLS* classical, *MES* mesenchymal, *PN* proneural. Significance was assessed via Tukey’s range test. NS indicates the test is not significant at *p* = 0.05. **b** Kaplan–Mayer survival curves, based on LGG TCGA RNA-seq for which survival information is available (*n* = 363 cases). Gene expression was averaged over the blood-derived and microglial signature genes, respectively, to assign a signature score to each case. The median signature score was used to divide cases into high-expressing and low-expressing cohorts. All comparisons were adjusted for age and gender, using cox proportional-hazards regression. *HR* hazard ratio

We calculated scores for blood-derived and microglial-TAM signature genes by averaging gene sets in glioma RNA-seq data from TCGA (*n* = 558). It is well-known that the degree of macrophage infiltration in glioma correlates with tumor grade [36]. However, this conclusion is based on studies that do not distinguish between macrophages of different lineages. Our data show a significant increase in blood-derived TAMs, but not in microglial TAMs, in GBM compared to LGG (Fig. 5a). In fact, astrocytomas have a degree of microglia infiltration, which is significantly higher than oligodendrogliomas or even GBMs (Tukey’s range test *p* < 0.01).

Survival analysis, corrected for age and gender, revealed that the signature of blood-derived TAMs correlates with significantly shorter survival in LGG (*p* = 0.016, hazard ratio [HR] = 3.44). However, there is no correlation between survival and the microglial-TAM signature (Fig. 5b). A similar correlation between blood-derived TAMs and survival is present in GBM (Additional file 2: Figure S5), although it is not significant at the 5% level (*p* = 0.109, HR = 1.61).

### A significant fraction of TAMs co-express canonical markers of M1 and M2 activation in individual cells

**Fig. 6:**
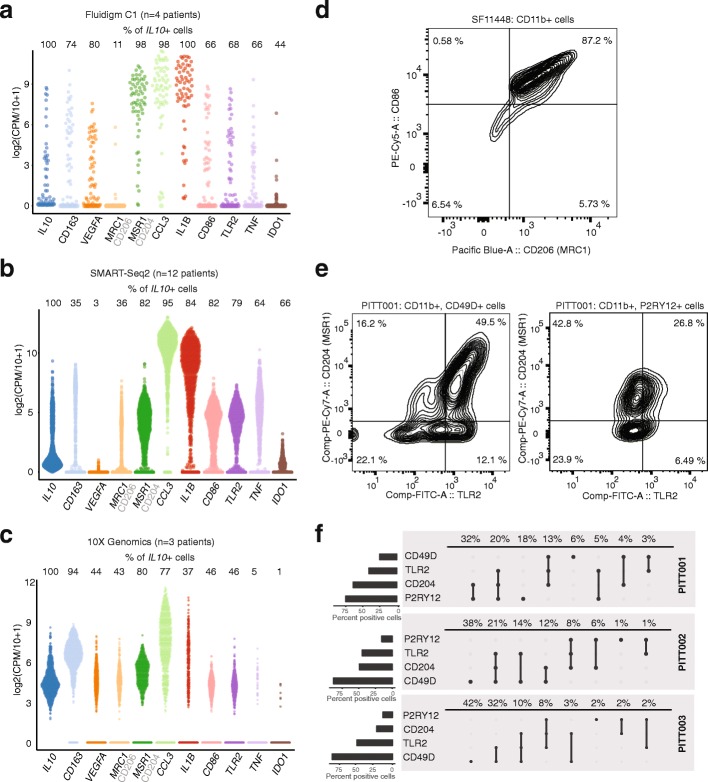
TAMs simultaneously co-express canonical M1 and M2 markers in individual cells. **a**–**c** Distributions of canonical M1 and M2 marker genes, in cells expressing *IL10*, compared across scRNA-seq platforms. **d** Flow cytometric analysis of tumor-infiltrating CD206 + CD86+ TAMs gated on live CD11b + myeloid cells. **e** Representative flow cytometric analysis of tumor-infiltrating CD204+ TLR2+ cells gated on CD11b+, CD49D+ live macrophages (*left*) and CD11b+, P2RY12+ live microglia (*right*). **f** Quantification of flow cytometric analysis. Cells positive for indicated markers are given by *circles* (*n* = 3 patients). The fraction of cells positive for each individual marker is given by the histogram on the *left*, the fraction of cells positive for each marker combination is given on top of each panel

As noted, we observed an increased expression of *IL10*, *TGFB2*, and genes associated with an oxidative metabolism in blood-derived TAMs, relative to microglia (Additional file 7: Table S6). These are all markers of macrophage M2 activation. Unexpectedly, however, we also found that individual TAMs frequently co-expressed canonical markers of both M1 and M2 activation (Fig. 6a–c). For example, in our C1-based scRNA-seq data, 66% of TAMs that express the M2 marker *IL10* also express the M1 marker TNF-α. We observed these non-canonical states in our TAM data from all three platforms, as well as in published scRNA-seq of TAMs derived from human melanoma samples (Additional file 2: Figure S6a) [37].

To further evaluate co-expression of M1 and M2 genes at the protein level, we performed analytical flow cytometry for CD11b, the M1 marker CD86, and the M2 marker CD206 (encoded by *MRC1*) in a GBM patient biopsy (SF11448). Consistent with our analysis on the messenger RNA (mRNA) level, we found a significant fraction of TAMs co-expressing these markers (Fig. 6d). To determine if these non-canonical states were restricted to a lineage, we then performed analytical flow cytometry on three additional GBM patient biopsies (PITT001, PITT002, PITT003). In addition to CD11b, P2RY12, and CD49D, we stained for TLR2 (a canonical M1 marker) and CD204 (encoded by *MSR1*, an M2 marker). In agreement with our scRNA-seq data, we found that the M1 and M2 markers were frequently co-expressed in individual CD11b + cells (Fig. 6e). Both P2RY12+ microglial TAMs and CD49D+ blood-derived TAMs demonstrated non-canonical states (Fig. 6f, Additional file 2: Figure S6b).

## Discussion

Our primary finding is that while blood-derived TAMs do significantly infiltrate pre-treatment human gliomas, they do not adopt the phenotype or regional distribution of microglial TAMs. Compared to microglia, blood-derived TAMs upregulate immunosuppressive cytokines, markers of active phagocytosis, and markers of an activated TCA cycle. To derive this result, we performed scRNA-seq of clinical glioma specimen. This uniquely enabled us to quantify differences between subpopulations of TAMs, in vivo. Our scRNA-seq identified a novel gene signature that distinguishes blood-derived macrophages from microglia in both malignant and non-malignant conditions. We mapped this signature to RNA-seq of microdissections from defined glioma anatomical structures. From that mapping, we show that microglia are enriched in the leading edge of tumor infiltration, while blood-derived TAMs are enriched near blood vessels and necrotic foci. The gene signature of blood-derived TAMs significantly and negatively correlates with survival in LGG, but the microglial-TAM signature does not. Collectively, these results support the idea that there are durable gene markers of macrophage lineage and that macrophage ontogeny is critical to shaping macrophage activation in the glioma microenvironment.

CX3CR1 is frequently used to identify microglia in tumor specimen [24]. However, we and others have found that purinergic receptors (e.g. P2RY12) are more specific than CX3CR1, as markers of microglial TAMs [9, 16, 17]. We present here a comprehensive list of markers to isolate TAMs by ontogeny, from human and mouse gliomas.

Venteicher et al. observed clear signatures for microglial and blood-derived macrophages in a PCA of TAMs from LGG. In addition, they found a continuum of intermediate transcriptional programs, rather than a bimodal distribution [17]. Two factors enabled us to develop a 66-gene signature, which separates TAMs by ontogeny in both malignant (Fig. 3a, Additional file 2: Figure S3b) and non-malignant (Fig. 4) tissues, both in human and in mouse (Fig. 4).

First, it is well-known that there is more macrophage infiltration in GBM compared to LGG. Our analyses support this being due to increased BMDM induction (Fig. 5a). Consistent with this, < 20% of the 1274 TAMs sequenced in Venteicher et al.’s study of LGG were BMDM, by our estimates. The TAMs we sequenced in this study are primarily from GBMs and approximately 70% of the 4181 novel TAMs profiled are BMDM. Thus, the combination dataset provides a comprehensive sampling of TAMs from both ontogenies and across glioma grades.

Second, the results of the murine lineage tracing experiments of Bowman et al. [9] were critically important as a basis for feature selection, before blood-derived vs microglial TAM classification. There is only a 5% overlap in a Gaussian mixture model of PC1 scores (Fig. 3a), using these genes. Moreover, the lineage-specific genes we identified tightly correlate by ontogeny and anti-correlate between ontogenies, across single cells (Fig. 3a, b), across the glioma population in both LGG and GBM cases (Fig. 3e), and across tumor regions (Fig. 3g). Thus, we conclude that this 66-gene signature distinguishes macrophages by ontogeny in both human and murine tissues, in both malignant and non-malignant conditions (Fig. 4).

Historically, macrophage activation has been classified into either a pro-inflammatory M1 state or an M2 state associated with the resolution of inflammation [38]. A more recent scRNA-seq study, in a mouse model of CNS injury, showed that macrophages can simultaneously express markers of both M1 and M2 activation [39]. Transcriptomic profiling of TAMs has shown limited overlap between TAM expression signatures and canonical M1/M2 expression profiles [40]. Furthermore, there are reports of M1 markers both positively [41] and negatively [24] correlating with glioma growth. In our data, individual TAMs co-express canonical markers of M1 and M2 activation with significant frequency, which may help explain these conflicting findings.

One limitation of our study is that expression at the protein level may not reflect expression at the mRNA level for all of our lineage markers derived from scRNA-seq. While we have validated several lineage markers at the protein level, which combination of these markers will be optimal for isolating TAMs by ontogeny, prospectively from human gliomas, is yet to be determined. Moreover, in this study we found that blood-derived TAMs adopt phenotypes that are distinct from those adopted by brain-resident microglia. Additional functional studies will be required to determine the mechanisms by which differences in ontogeny contribute to macrophage activation toward M1 or M2.

## Conclusion

There is mounting evidence that systemic immune activation is required for an optimal anti-tumor response [42]. To what extent peripheral BMDM contribute to the TAM pool and how macrophage ontogeny shapes macrophage activation is therefore of critical importance to the development of immunotherapies. We used scRNA-seq, combined with public data meta-analysis, to show that blood-derived and microglial TAMs exhibit distinct phenotypes and distinct localizations within the tumor. Blood-derived TAMs upregulate M2-associated immunosuppressive cytokines and markers of an oxidative metabolism that are characteristic of the M2 phenotype. These results argue against status quo approaches which target both lineages equally and in favor of strategies to specifically deplete the immunosuppressive blood-derived fraction. To the best of our knowledge, this work represents the first application of scRNA-seq to GBM-derived myeloid cells. Both the data and results presented here will enable future studies of the effect of therapy on the immune response, by contributing to our baseline knowledge of innate immunity in untreated glioma.

## Methods

### Tumor tissue acquisition and processing

We acquired fresh tumor tissue from patients undergoing surgical resection for glioma. De-identified samples were provided by the Neurosurgery Tissue Bank at the University of California San Francisco (UCSF). Sample use was approved by the Institutional Review Board at UCSF. The experiments performed here conform to the principles set out in the WMA Declaration of Helsinki and the Department of Health and Human Services Belmont Report. All patients provided informed written consent. Tissues were minced in collection media (Leibovitz’s L-15 medium, 4 mg/mL glucose, 100 u/mL Penicillin, 100 ug/mL Streptomycin) with a scalpel. Samples dissociation was carried out in a mixture of papain (Worthington Biochem. Corp) and 2000 units/mL of DNase I freshly diluted in EBSS and incubated at 37 °C for 30 min. After centrifugation (5 min at 300 g), the suspension was resuspended in PBS. Subsequently, suspensions were triturated by pipetting up and down ten times and then passed through a 70-μm strainer cap (BD Falcon). Last, centrifugation was performed for 5 min at 300 g. After resuspension in PBS, pellets were passed through a 40-μm strainer cap (BD Falcon), followed by centrifugation for 5 min at 300 g. The dissociated, single cells were then resuspended in GNS (Neurocult NS-A (Stem Cell Tech.), 2 mM L-Glutamine, 100 U/mL Penicillin, 100 ug/mL Streptomycin, N2/B27 supplement (Invitrogen), sodium pyruvate).

### CD11b + cell isolation

In total, 20 μL of CD11b microbeads (Miltenyi Biotec; 130-093-634) were mixed with the 80 μL single-cell suspension (produced as above) in PBS supplemented with 2 μM EDTA and 0.5% bovine serum albumin (BSA) (MACS buffer) and incubated at 4 °C for 15 min. Cells were washed twice with MACs buffer, centrifuged for 10 min at 300 g, and resuspended in MACs buffer. The suspension was then applied to a MACS LS column in the magnetic field of a MACS Separator. Columns were washed three times with MACs buffer and magnetically labeled cells were then flushed into a collection tube. The purity of CD11b + cells was assessed via flow cytometry: CD11b + and CD11b– fractions were staining with phycoerythrin-conjugated anti-CD11b (Clone M1/70) antibody for 15 min; cells were then washed twice and analyzed on a FACsCaliber flow cytometer using FACSDIVA software (Additional file 2: Figure S1a).

### Single-cell RNA sequencing

#### Fluidigm C1-based scRNA-seq

Fluidigm C1 Single-Cell Integrated Fluidic Circuit (IFC) and SMARTer Ultra Low RNA Kit were used for single-cell capture and complementary DNA (cDNA) generation. cDNA quantification was performed using Agilent High Sensitivity DNA Kits and diluted to 0.15–0.30 ng/μL. The Nextera XT DNA Library Prep Kit (Illumina) was used for dual indexing and amplification with the Fluidigm C1 protocol. Ninety-six scRNA-seq libraries were generated from each tumor/Cd11b + sample and subsequently pooled for 96-plex sequencing. cDNA was purification and size selection was carried out twice using 0.9X volume of Agencourt AMPure XP beads (Beckman Coulter). The resulting cDNA libraries were quantified using High Sensitivity DNA Kits (Agilent).

#### 10X genomics-based scRNA-seq

Tissue was dissociated by incubation in papain with 10% DNAse for 30 min. A single-cell suspension was obtained by manual trituration using a glass pipette. The cells were filtered via an ovomucoid gradient to remove debris, pelleted, and resuspended in Neural Basal Media with serum at a concentration of 1700 cells/uL. In total, 10.2 uL of cells were loaded into each well of a 10X Chromium Single Cell capture chip and a total of two lanes were captured. Single-cell capture, reverse transcription, cell lysis, and library preparation were performed per manufacturer’s protocol.

Sequencing for both platforms was performed on a HiSeq 2500 (Illumina, 100-bp paired-end protocol).

### Exome-sequencing and genomic mutation identification

The NimbleGen SeqCap EZ Human Exome Kit v3.0 (Roche) was used for exome capture on a tumor sample and a blood control sample from each patient. Samples were sequenced with an Illumina-HiSeq 2500 machine (100-bp paired-end reads). Reads were mapped to the human grch37 genome with BWA [43] and only uniquely matched paired reads were used for analysis. PicardTools (http://broadinstitute.github.io/picard/) and the GATK toolkit [44] carried out quality score re-calibration, duplicate-removal, and re-alignment around indels. Large-scale (>100 Exons) somatic copy number variants (CNVs) were inferred with ADTex [45]. To increase CNV size, proximal (< 1 Mbp) CNVs were merged. Somatic SNVs were inferred with MuTect (https://www.broadinstitute.org/cancer/cga/mutect) for each tumor/control pair and annotated with the Annovar software package [46].

### Single-cell RNA-sequencing data processing and neoplastic-cell classification

Data processing was performed as described previously [14]. Briefly, reads were quality trimmed and TrimGalore! (http://www.bioinformatics.babraham.ac.uk/projects/trim_galore/) clipped Nextera adapters. HISAT2 [47] was used to perform alignments to the grch37 human genome. Gene expression was quantified using the ENSEMBL reference with featureCounts [48]. Only correctly paired, uniquely mapped reads were kept. In each cell, expression values were scaled to counts per million (CPM). Low-quality cells were filtered by thresholding number of genes detected at 800 and at least 50,000 uniquely aligned reads. tSNE plots visualizing groupings of cells were carried out using the Seurat R package [49]. CNVs that were called in matched exome-seq data were quantified in individual cells as previously described [15]. Briefly, megabase-scale CNVs were identified in tumor/normal paired exome-seq datasets, and then quantified in individual cells using a control sample from non-malignant brain.

### Public data acquisition

Expression matrices from bulk RNA-seq (performed in triplicate) were downloaded from GEO for the following samples: representing BMDM, we obtained M0 (GSE68482) [50], M1, M2 macrophages (GSE36952) [51], and monocytes (GSE58310) [52]. We also obtained data for microglia purified from epilepsy-related surgical specimen (*n* = 3) and post-mortem brain (*n* = 5) (GSE80338) [53]. Lists of genes that are differentially expressed between blood-derived murine TAMs and microglial murine TAMs, in two murine glioma models, were downloaded [9]. Normalized scRNA-seq counts were obtained from GEO for astrocytoma (GSE89567) and oligodendroglioma (GSE70630). Analysis was restricted to TAMs, as classified in the BROAD single-cell data portal (https://portals.broadinstitute.org/single_cell). Normalized counts from TCGA RNA-seq data were obtained from the Genomics Data Commons portal (https://gdc.cancer.gov/). Patients diagnosed as GBM and wild-type IDH1 expression (*n* = 144) as well as those with LGG classification and IDH1 mutation (*n* = 414), as given in [54], were normalized to log2(CPM + 1) and used for analysis. Z-score normalized counts from regional RNA-seq of 122 samples from ten patients was obtained via the web interface of the IVY GAP (http://glioblastoma.alleninstitute.org/) database. Furthermore, images of in situ RNA hybridizations in glioma tissue sections were downloaded for two patients: *BIN1*: W11-1-1-E.1.04, 57-year-old man, glioblastoma; *TGFBI*: W8-1-1-B.1.04, 49-year-old woman, glioblastoma.

### Derivation of ontogeny-specific expression signatures

Genes differentially expressed between blood-derived TAMs and microglial TAMs, recurrently in both of Bowman et al.’s two murine glioma models, were used as a starting point [9]. We identified homologues of these differentially expressed mouse genes with the biomaRt package in R [55]. The resulting set of genes was filtered for genes expressed in our human-TAM scRNA-seq data. Genes with a mean expression > 1 CPM were retained. This set of genes was used as the basis for subsequent PCA and single-cell consensus clustering (SC3). Expression values, defined as log2 (CPM/10 + 1), of genes in the human-TAM scRNA-seq data were z-score normalized, across cells from within each single-cell platform (SMARTer vs SMART-Seq2) independently. Subsequently, PCA followed by Varimax rotation was performed. Sample scores, along PC1, were partitioned using a two-component Gaussian mixture model. Genes strongly associated with PC1 in either direction were identified by applying a threshold of abs(loading) > 0.2 to the gene loadings. MFA was performed on the Smart-Seq2 and C1 data, using the FactoMineR (https://cran.r-project.org/web/packages/FactoMineR/index.html) R package, using the 237 mouse homologue genes. Genes strongly loading PC1 in the PCA were compared to RNA-seq data from microdissections of defined glioma anatomical structures, via the IVY atlas (http://glioblastoma.alleninstitute.org/), and visualized with morpheus (https://software.broadinstitute.org/morpheus/). SC3 clustering [56] (k = 2) was also performed in the human-TAM scRNA-seq data, restricted to the set of human counterparts of lineage-specific murine-TAM genes. Both classifiers produced highly similar classification results (Matthews correlation coefficient = 0.946). To identify genes significantly co-occurring in single cells, we calculated the odds ratios (OR) and *p* values as described in [57]. *P* values were corrected for multiple testing with Benjamini–Hochberg.

### Calculation of ontogeny scores and survival analysis

For each sample in the TCGA dataset (described above) we calculated the average expression of microglial-TAM genes and blood-derived TAM genes, respectively. To compare the relative amount of infiltration between glioma subtypes, we utilized the glioVis portal [58] to classify isocitrate dehydrogenase 1/2 (IDH1/2) wild-type GBM samples into three transcriptional subtypes: Classical; Mesenchymal; and Proneural. IDH1/2-mutant LGGs were subdivided into astrocytomas (n = 110) and oligodendrogliomas (n = 117) based on histology and the presence/absence of a 1p/19p co-deletion.

For both the microglial and blood-derived TAM survival analysis, Progene V2 was used. High- and low-expression cohorts were defined as cases with expression scores above and below the median score, respectively. GBMs and LGGs were considered separately. We adjusted for age and gender by adding these covariates to a cox proportional hazards model [59].

### Analytical flow cytometry

De-identified fresh glioma tissues were obtained as described above, in “Tumor tissue acquisition and processing.” Tissue was mechanically dissociated, resuspended in 70% Percoll (Sigma-Aldrich), overlaid with 37% and 30% Percoll, and centrifuged for 20 min at 500 × g. Enriched leukocyte populations (TIL) were recovered at the 70–37% interface, washed twice in PBS, and resuspended in flow staining buffer (PBS + 1% BSA) containing Human TruStain FcX (Biolegend). Cells were then incubated at 4° for 30 min with antibodies, washed twice in flow staining buffer, and analyzed on a BD FACSAria cell sorter.

The following antibodies were purchased from Biolegend: FITC anti-mouse/human CD282; PE anti-human P2RY12; PE/Cy7 anti-human CD204; APC/Fire™ 750 anti-mouse/human CD11b; APC anti-human CD49d; PerCP/Cy5.5 anti-human HLA-DR; and BV421 anti-human CD206. All antibodies were used according to the manufacturers’ recommended usage.

## Additional files

Additional file 1: Table S1. Sample overview. Relevant clinical information and histologic assessment of samples included in the study. (XLSX 10 kb)

Additional file 2: Supplementary Figures, Supplementary figures and legends. (PDF 3980 kb)

Additional file 3: Table S2. Markers of ontogeny in murine TAMs, compared to human macrophages. Genes differentially expressed between blood-derived TAMs and microglial TAMs in mouse (column A), their homologues in human (column B), and their intersection with genes expressed by human macrophages/microglia in nonmalignant (column C) and malignant (column D) tissue. (XLSX 34 kb)

Additional file 4: Table S3. PCA loadings for human TAMs. Loadings of the 237 genes used in the PCA of Fig. 3a. (XLSX 22 kb)

Additional file 5: Table S4. Markers of macrophage ontogeny. The 66 lineage-specific genes identified in Fig. 3. (XLSX 9 kb)

Additional file 6: Table S5. PCA loadings for human TAMs compared to macrophages/microglia from non-malignant tissues. Genes and their loadings in the PCA of Fig. 4. (XLSX 12 kb)

Additional file 7: Table S6. Genes differentially expressed between human blood-derived and microglial TAMs. The results of a differential-expression test between human blood-derived and microglial TAMs, performed via DESeq. (XLSX 1113 kb)

## Abbreviations

ASTRO: Astrocytoma
BMDM: Bone marrow-derived macrophage
CLS: Classical
CNS: Central nervous system
CPM: Counts per million
GBM: Glioblastoma
IGAP: Ivy Glioblastoma Atlas Project
LGG: Low-grade glioma
M1: Classically activated macrophage
M2: Alternatively activated macrophage
MES: Mesenchymal
OLIG: Oligodendroglioma
PC1: Principal component 1
PCA: Principal components analysis
PN: Proneural
RNA-seq: RNA sequencing
scRNA-seq: Single-cell RNA sequencing
SMART: Switching Mechanism at 5’ End of RNA Template
TAM: Tumor-associated macrophage
TCA: Tricarboxylic acid
TCGA: The Cancer Genome Atlas
tSNE: t-distributed stochastic neighbor embedding

## References

[1] Pyonteck SM, Akkari L, Schuhmacher AJ, Bowman RL, Sevenich L, Quail DF (2013). CSF-1R inhibition alters macrophage polarization and blocks glioma progression. Nat Med.

[2] Butowski N, Colman H, De Groot JF, Omuro AM, Nayak L, Wen PY (2016). Orally administered colony stimulating factor 1 receptor inhibitor PLX3397 in recurrent glioblastoma: An Ivy Foundation Early Phase Clinical Trials Consortium phase II study. Neuro Oncol.

[3] Joyce JA, Quail DF (2017). Molecular pathways: deciphering mechanisms of resistance to macrophage-directed therapies. Clin Cancer Res.

[4] Castro BA, Flanigan P, Jahangiri A, Hoffman D, Chen W, Kuang R (2017). Macrophage migration inhibitory factor downregulation: a novel mechanism of resistance to anti-angiogenic therapy. Oncogene.

[5] Ginhoux F, Lim S, Hoeffel G, Low D, Huber T (2013). Origin and differentiation of microglia. Front Cell Neurosci.

[6] Zhou W, Ke SQ, Huang Z, Flavahan W, Fang X, Paul J (2015). Periostin secreted by glioblastoma stem cells recruits M2 tumour-associated macrophages and promotes malignant growth. Nat Cell Biol.

[7] Hambardzumyan D, Gutmann DH, Kettenmann H (2015). The role of microglia and macrophages in glioma maintenance and progression. Nat Neurosci.

[8] Müller A, Brandenburg S, Turkowski K, Müller S, Vajkoczy P (2015). Resident microglia, and not peripheral macrophages, are the main source of brain tumor mononuclear cells. Int J Cancer.

[9] Bowman RL, Klemm F, Akkari L, Pyonteck SM, Sevenich L, Quail DF (2016). Macrophage ontogeny underlies differences in tumor-specific education in brain malignancies. Cell Rep.

[10] Muller S, Kohanbash G, Liu J, Alvarado B, Carrera D, Bhaduri A, et al. Single-cell profiling maps the spectrum of crosstalk between glioma cells and tumor associated macrophages. European Genome-Phenome Archive. https://www.ebi.ac.uk/ega/studies/EGAS00001002185

[11] Muller S, Liu J, Di Lullo E, Malatesta M, Pollen A, Nowakowski T, et al. Comparison of EGF and PDGF driven glioblastomas. European Genome-Phenome Archive. https://www.ebi.ac.uk/ega/studies/EGAS00001001900

[12] Tirosh I, Suva M. Single cell RNA-seq analysis of IDH-mutant astrocytoma. Gene Expression Omnibus. https://www.ncbi.nlm.nih.gov/geo/query/acc.cgi?acc=GSE89567

[13] Tirosh I, Suva M. Single cell RNA-seq analysis of oligodendroglioma. Gene Expression Omnibus. https://www.ncbi.nlm.nih.gov/geo/query/acc.cgi?acc=GSE70630

[14] Diaz A, Liu SJ, Sandoval C, Pollen A, Nowakowski TJ, Lim DA (2016). SCell: integrated analysis of single-cell RNA-seq data. Bioinformatics.

[15] Müller S, Liu SJ, Di Lullo E, Malatesta M, Pollen AA, Nowakowski TJ (2016). Single‐cell sequencing maps gene expression to mutational phylogenies in PDGF‐ and EGF‐driven gliomas. Mol Syst Biol.

[16] Müller S, Diaz A (2017). Single-Cell mRNA sequencing in cancer research: integrating the genomic fingerprint. Front Genet.

[17] Venteicher AS, Tirosh I, Hebert C, Yizhak K, Neftel C, Filbin MG (2017). Decoupling genetics, lineages, and microenvironment in IDH-mutant gliomas by single-cell RNA-seq. Science.

[18] Zhang W, Duan S. High salt primes a specific activation state of macrophages, M(Na). Gene Expression Omnibus. https://www.ncbi.nlm.nih.gov/geo/query/acc.cgi?acc=GSE68482 10.1038/cr.2015.87 PMC4528058 26206316

[19] Mallmann B, Schultze J. Transcriptome wide analysis of classically and alternatively activated macrophages. Gene Expression Omnibus. https://www.ncbi.nlm.nih.gov/geo/query/acc.cgi?acc=GSE36952

[20] Saeed S, Quintin J, Rao N, Kerstens H, Aghajanirefah A, Matarese F. Epigenetic programming during monocyte to macrophage differentiation and trained innate immunity. Gene Expression Omnibus. https://www.ncbi.nlm.nih.gov/geo/query/acc.cgi?acc=GSE58310 10.1126/science.1251086 PMC4242194 25258085

[21] Szulzewsky F, Arora S. Human glioblastoma-associated microglia/monocytes express a distinct RNA profile compared to human control and murine samples. Gene Expression Omnibus. https://www.ncbi.nlm.nih.gov/geo/query/acc.cgi?acc=GSE80338 10.1002/glia.23014 27312099

[22] TCGA Research Network. The Cancer Genome Atlas. https://cancergenome.nih.gov

[23] Kierdorf K, Erny D, Goldmann T, Sander V, Schulz C, Gomez Perdiguero E (2013). Microglia emerge from erythromyeloid precursors via Pu.1- and Irf8-dependent pathways. Nat Neurosci.

[24] Feng X, Szulzewsky F, Yerevanian A, Chen Z, Heinzmann D, Rasmussen RD (2015). Loss of CX3CR1 increases accumulation of inflammatory monocytes and promotes gliomagenesis. Oncotarget.

[25] Geissmann F, Jung S, Littman DR (2003). Blood monocytes consist of two principal subsets with distinct migratory properties. Immunity.

[26] Ginhoux F, Greter M, Leboeuf M, Nandi S, See P, Mehler MF (2013). Fate mapping analysis reveals that adult microglia derive from primitive macrophages. Science.

[27] Bennetta ML, Bennetta C, Liddelowa SA, Ajami B, Zamanian JL, Fernhoff NB (2016). New tools for studying microglia in the mouse and human CNS. Proc Natl Acad Sci U S A.

[28] Butovsky O, Jedrychowski MP, Moore CS, Cialic R, Lanser AJ, Gabriely G (2014). Identification of a unique TGF-β-dependent molecular and functional signature in microglia. Nat Neurosci.

[29] Foltz G, Pulchalski R, Shah N. Ivy Glioblastoma Atlas Project. http://glioblastoma.alleninstitute.org

[30] Goldmann T, Wieghofer P, Joana M, Jordão C, Prutek F, Hagemeyer N (2016). Origin, fate and dynamics of macrophages at central nervous system interfaces. Nat Immunol.

[31] Darmanis S, Sloan SA, Zhang Y, Enge M, Caneda C, Shuer LM (2015). A survey of human brain transcriptome diversity at the single cell level. Proc Natl Acad Sci.

[32] Williams K, Alvarez X, Lackner A (2001). Central nervous system perivascular cells are immunoregulatory cells that connect the CNS with the peripheral immune system. Glia.

[33] Wehner T, Klett FF, Bechmann I, Priller J, Kovac A, Bo M (2001). Immune surveillance of mouse brain perivascular spaces by blood-borne macrophages. Eur J Neurosci.

[34] Tannahill GM, Curtis AM, Adamik J, Mcgettrick AF, Goel G, Frezza C (2013). Succinate is an inflammatory signal that induces IL-1β through HIF-1α. Nature.

[35] Jha AK, Huang SC, Sergushichev A, Lampropoulou V, Ivanova Y, Loginicheva E (2015). Network integration of parallel metabolic and transcriptional data reveals metabolic modules that regulate macrophage polarization. Immunity.

[36] Roggendorf W, Strupp S, Paulus W (1996). Distribution and characterization of microglia/macrophages in human brain tumors. Acta Neuropathol.

[37] Tirosh I, Izar B, Prakadan SM, Wadsworth MH, Treacy D, Trombetta JJ (2016). Dissecting the multicellular ecosystem of metastatic melanoma by single-cell RNA-seq. Science.

[38] Mantovani A, Sozzani S, Locati M, Allavena P, Sica A (2002). Macrophage polarization: Tumor-associated macrophages as a paradigm for polarized M2 mononuclear phagocytes. Trends Immunol.

[39] Kim K-T, Lee HW, Lee H-O, Song HJ, Jeong DE, Shin S (2016). Application of single-cell RNA sequencing in optimizing a combinatorial therapeutic strategy in metastatic renal cell carcinoma. Genome Biol.

[40] Szulzewsky F, Pelz A, Feng X, Synowitz M, Markovic D, Langmann T (2015). Glioma-associated microglia/macrophages display an expression profile different from M1 and M2 polarization and highly express Gpnmb and Spp1. PLoS One.

[41] Zeiner PS, Preusse C, Blank A-E, Zachskorn C, Baumgarten P, Caspary L (2015). MIF receptor CD74 is restricted to microglia/macrophages, associated with a M1-polarized immune milieu and prolonged patient survival in gliomas. Brain Pathol.

[42] Spitzer MH, Carmi Y, Reticker-Flynn NE, Kwek SS, Madhireddy D, Martins MM (2017). Systemic immunity is required for effective cancer immunotherapy. Cell.

[43] Li H, Durbin R (2009). Fast and accurate short read alignment with Burrows-Wheeler transform. Bioinformatics.

[44] McKenna A, Hanna M, Banks E, Sivachenko A, Cibulskis K, Kernytsky A (2010). The Genome Analysis Toolkit: a MapReduce framework for analyzing next-generation DNA sequencing data. Genome Res.

[45] Amarasinghe KC, Li J, Hunter SM, Ryland GL, Cowin PA, Campbell IG (2014). Inferring copy number and genotype in tumour exome data. BMC Genomics.

[46] Wang K, Li M, Hakonarson H (2010). ANNOVAR: Functional annotation of genetic variants from high-throughput sequencing data. Nucleic Acids Res.

[47] Kim D, Pertea G, Trapnell C, Pimentel H, Kelley R, Salzberg SL (2013). TopHat2: accurate alignment of transcriptomes in the presence of insertions, deletions and gene fusions. Genome Biol.

[48] Liao Y, Smyth GK, Shi W (2014). FeatureCounts: An efficient general purpose program for assigning sequence reads to genomic features. Bioinformatics.

[49] Satija R, Farrell JA, Gennert D, Schier AF, Regev A (2015). Spatial reconstruction of single-cell gene expression data. Nat Biotechnol.

[50] Zhang W-C, Zheng X-J, Du L-J, Sun J-Y, Shen Z-X, Shi C (2015). High salt primes a specific activation state of macrophages, M(Na). Cell Res.

[51] Beyer M, Mallmann MR, Xue J, Staratschek-Jox A, Vorholt D, Krebs W (2012). High-resolution transcriptome of human macrophages. PLoS One.

[52] Saeed S, Quintin J, Kerstens HHD, Rao NA, Aghajanirefah A, Matarese F (2014). Epigenetic programming of monocyte-to-macrophage differentiation and trained innate immunity. Science.

[53] Szulzewsky F, Arora S, de Witte L, Ulas T, Markovic D, Schultze JL (2016). Human glioblastoma-associated microglia/monocytes express a distinct RNA profile compared to human control and murine samples. Glia.

[54] Ceccarelli M, Barthel FP, Malta TM, Sabedot TS, Salama SR, Murray BA (2016). Molecular profiling reveals biologically discrete subsets and pathways of progression in diffuse glioma. Cell.

[55] Durinck S, Spellman PT, Birney E, Huber W (2009). Mapping identifiers for the integration of genomic datasets with the R/Bioconductor package biomaRt. Nat Protoc.

[56] Kiselev VY, Kirschner K, Schaub MT, Andrews T, Yiu A, Chandra T (2017). SC3: consensus clustering of single-cell RNA-seq data. Nat Methods.

[57] Gao J, Aksoy BA, Dogrusoz U, Dresdner G, Gross B, Sumer SO (2013). Integrative analysis of complex cancer genomics and clinical profiles using the cBioPortal. Sci Signal.

[58] Bowman RL, Wang Q, Carro A, Verhaak RGW, Squatrito M (2017). GlioVis data portal for visualization and analysis of brain tumor expression datasets. Neuro Oncol.

[59] Goswami CP, Nakshatri H (2014). PROGgeneV2: enhancements on the existing database. BMC Cancer.

