# Supplementary material for: Single-cell profiling of human gliomas reveals macrophage ontogeny as a basis for regional differences in macrophage activation in the tumor microenvironment
Source: Genome Biol. 2017 Dec 20;18:234. doi: 10.1186/s13059-017-1362-4 (PMC5738907; doi:10.1186/s13059-017-1362-4)
Supplement: Supplementary file 2 — Supplementary Figures, Supplementary figures and legends. (PDF 3980 kb) [file 13059_2017_1362_MOESM2_ESM.pdf]

Supplementary Figures

Figure S1 – *ScRNA-seq data quality control.* (a) Fractions of CD11b-expressing cells (estimated *via* flow-cytometry) in suspensions derived from GBM biopsy SF10360. The top panel shows the residual following negative selection for CD11b using our microbead protocol, and contains only 1.33% CD11b+ cells. The bottom panel shows that 96.3% of cells positively selected using our microbead strategy express CD11b. (b) TSNE clustering of 997 cells from GBM biopsy SF11232. (c) Top panel: TSNE clustering of 14,562 cells from 3 cases (SF11136, SF11147, SF11232). Cells from each tumor are indicated by color. Bottom panel: Here color indicates expression level of canonical marker genes. (d) PCA (top) and TSNE clustering (bottom) of cells from LGG biopsy SF11136 from two independent 10x captures. (e) Distributions of gene expression for canonical markers, across 1,274 single cells from the 12 LGG cases of Venteicher *et al.* [17].

Figure S2 – *Platform-specific PCA of human TAMs.* (a) A PCA of TAMs from C1-based (top) and Smart-Seq2-based (bottom) scRNA-seq in the space of the 237 mouse homologues from Bowman *et al*. The density curves of a Gaussian mixture model are in gray. (b) Top: Multiple factor analysis of individuals (237 marker genes) from two groups (single cells from Smart-Seq2 and C1; variables). Individuals (genes) are represented by dots. Bottom: MFA table contributions for Smart-Seq2 and C1 data tables. (c) The distributions of cells from each platform, across the first principal component of the C1-Smart-seq2 combined PCA.

Figure S3 – *Extended analysis of the proposed signature genes.* (a) Hierarchical clustering of Pearson correlation coefficients, obtained via RNA-seq of LGGs and GBMs from TCGA (n=558 cases). (b) Top panel: PCA of human TAMs (n=3 patients, n=4,039 cells), in the space of genes that are differentially expressed between murine-TAM lineages. Cells positive for *P2RY12*, *SIGLEC8* or *NAV3* are indicated by red dots. Middle panel: Odds ratio (OR) for co-expression with *P2RY12*, based on 4,039 single cells from 3 patients. *=adjusted p-value <0.05 (Fisher's exact test). Bottom panel: Same as in the middle panel, but for co-expression with *TGFBI*. (c) Gene expression for TAM lineage markers in the combined C1/Smart-seq2 scRNA-seq data. Bar height

represents the mean and whisker length gives a 95% confidence interval. Genes are sorted from highest to lowest absolute log2 fold-change over sum of variance. (d) Gene expression in IVYGAP for markers of TAM ontogeny. Each column represents a gene, each row annotates expression in RNA-seq of an anatomically defined tumor compartment.

Figure S4 – *Pathway analysis of genes enriched in blood-derived TAMs relative to microglial TAMs***.** Pathways from the KEGG database (http://www.genome.jp), that are over-represented in genes up-regulated by blood-derived TAMs.

Figure S5 – *Over-expression of the signature genes of blood-derived TAMs, but not the signature of microglial TAMs, inversly correlates with survival***.** Kaplan-Mayer survival curves were generated based on RNA-seq of LGGs (n=363) and GBMs (n=138) from TCGA.

Figure S6 – *TAMs from human melanomas simultaniously express M1 and M2 markers in individual cells.* (a) Distributions of canonical M1 and M2 marker genes, in TAMs from human melanomas that express *IL10* (n=108 cells from 7 cases), library prep was based on SMART-Seq2. (b) Flow cytometric analysis of tumor-infiltrating CD204+TLR2+ cells gated on CD11b+, CD49D+ live macrophages (left) and CD11b+, P2RY12+ live microglia (right) from two GBM patient biopsies.

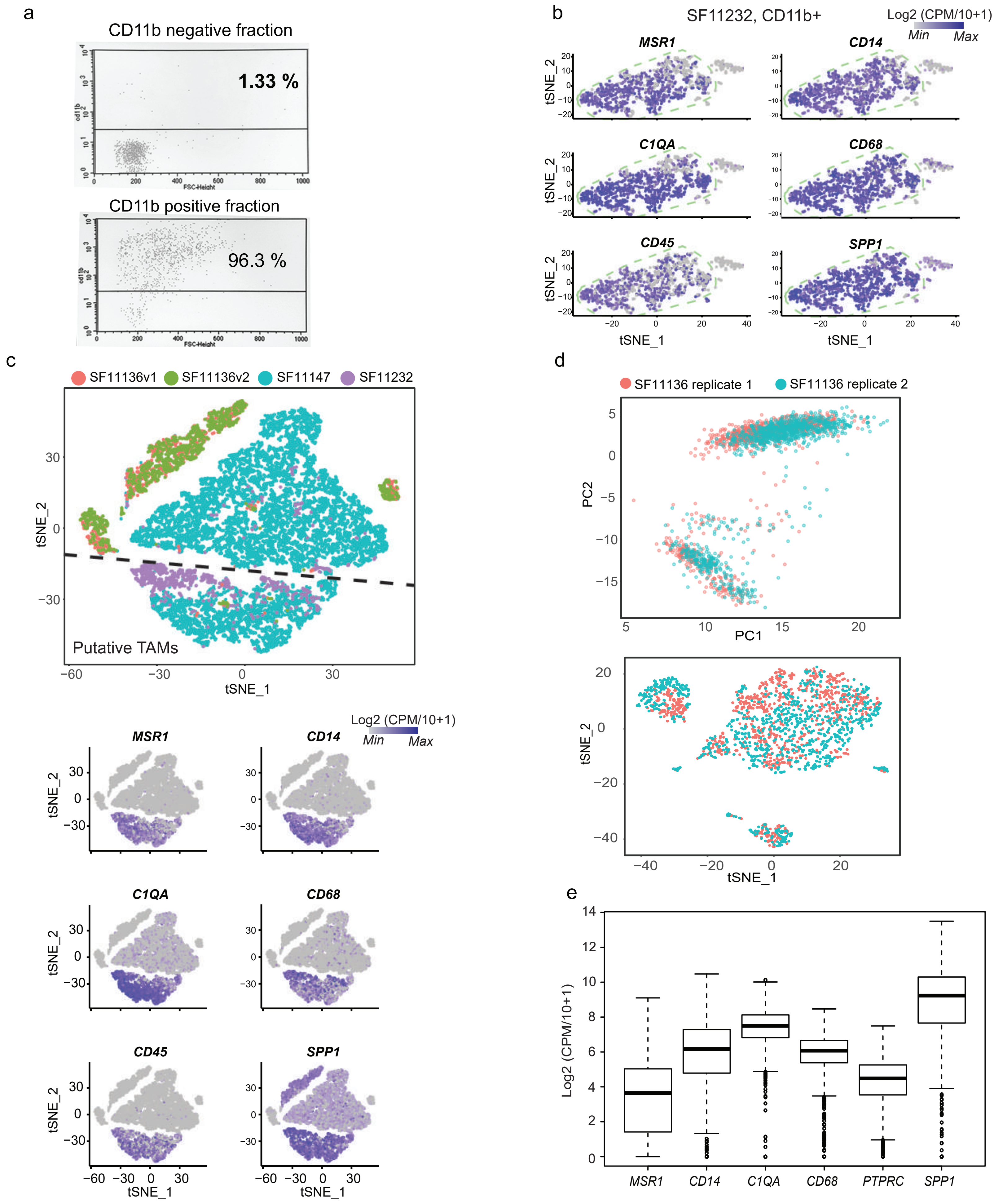
a
CD11b negative fraction
1.33 %
CD11b positive fraction
96.3 %
FSC-Height
b
SF11232, CD11b+
Log2 (CPM/10+1)
Min
Max
MSR1
CD14
C1QA
CD68
CD45
SPP1
tSNE_2
tSNE_1
c
SF11136v1
SF11136v2
SF11147
SF11232
Putative TAMs
d
SF11136 replicate 1
SF11136 replicate 2
PC1
PC2
e
Log2 (CPM/10+1)
MSR1
CD14
C1QA
CD68
PTPRC
SPP1

Figure S1

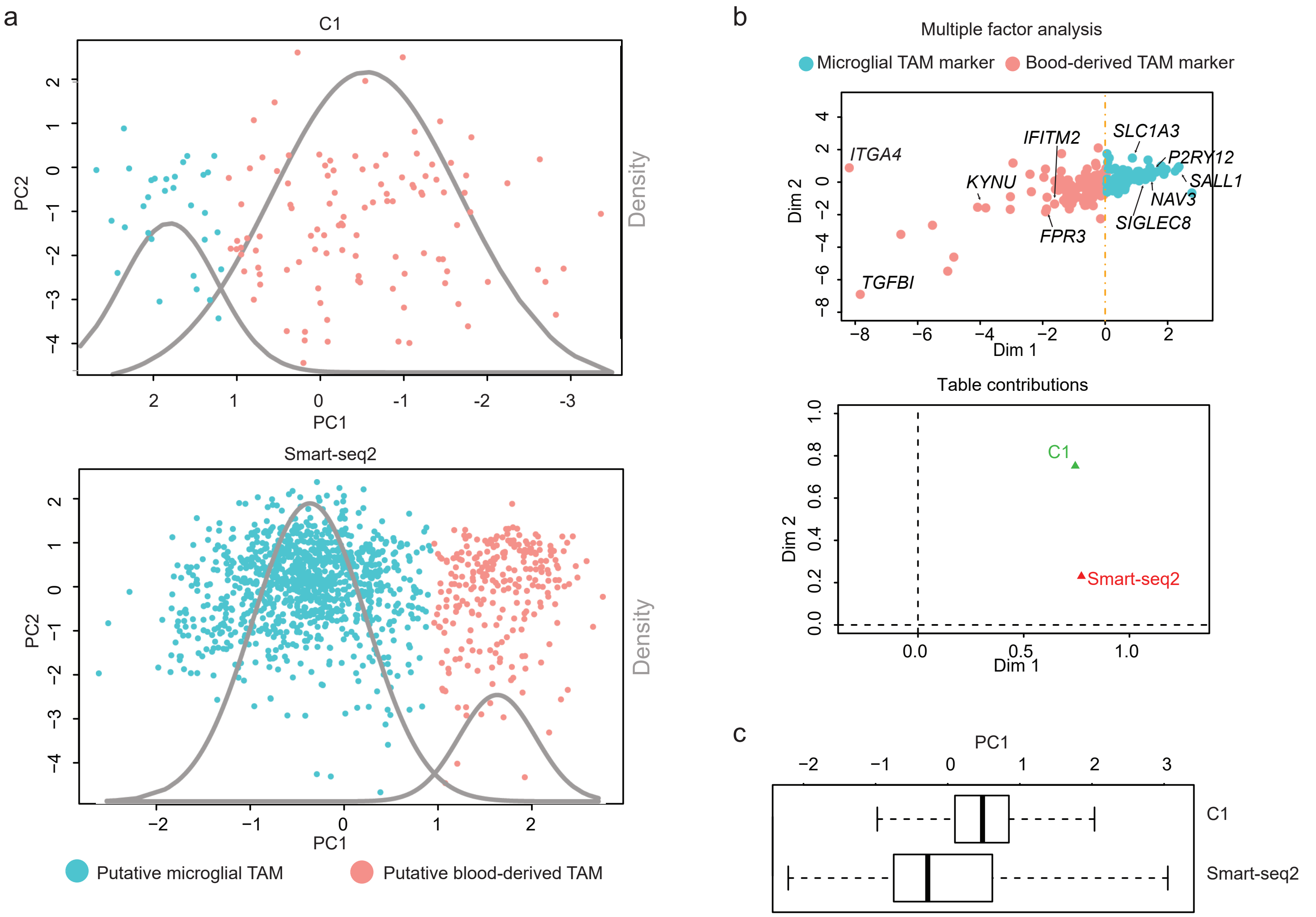

a
C1
PC2
PC1
Density
Smart-seq2
Putative microglial TAM
Putative blood-derived TAM
b
Multiple factor analysis
Microglial TAM marker
Bood-derived TAM marker
Dim 1
Dim 2
ITGA4
KYNU
IFITM2
FPR3
TGFBI
SLC1A3
P2RY12
SALL1
NAV3
SIGLEC8
Table contributions
C1
Smart-seq2
c
PC1
C1
Smart-seq2

Figure S2

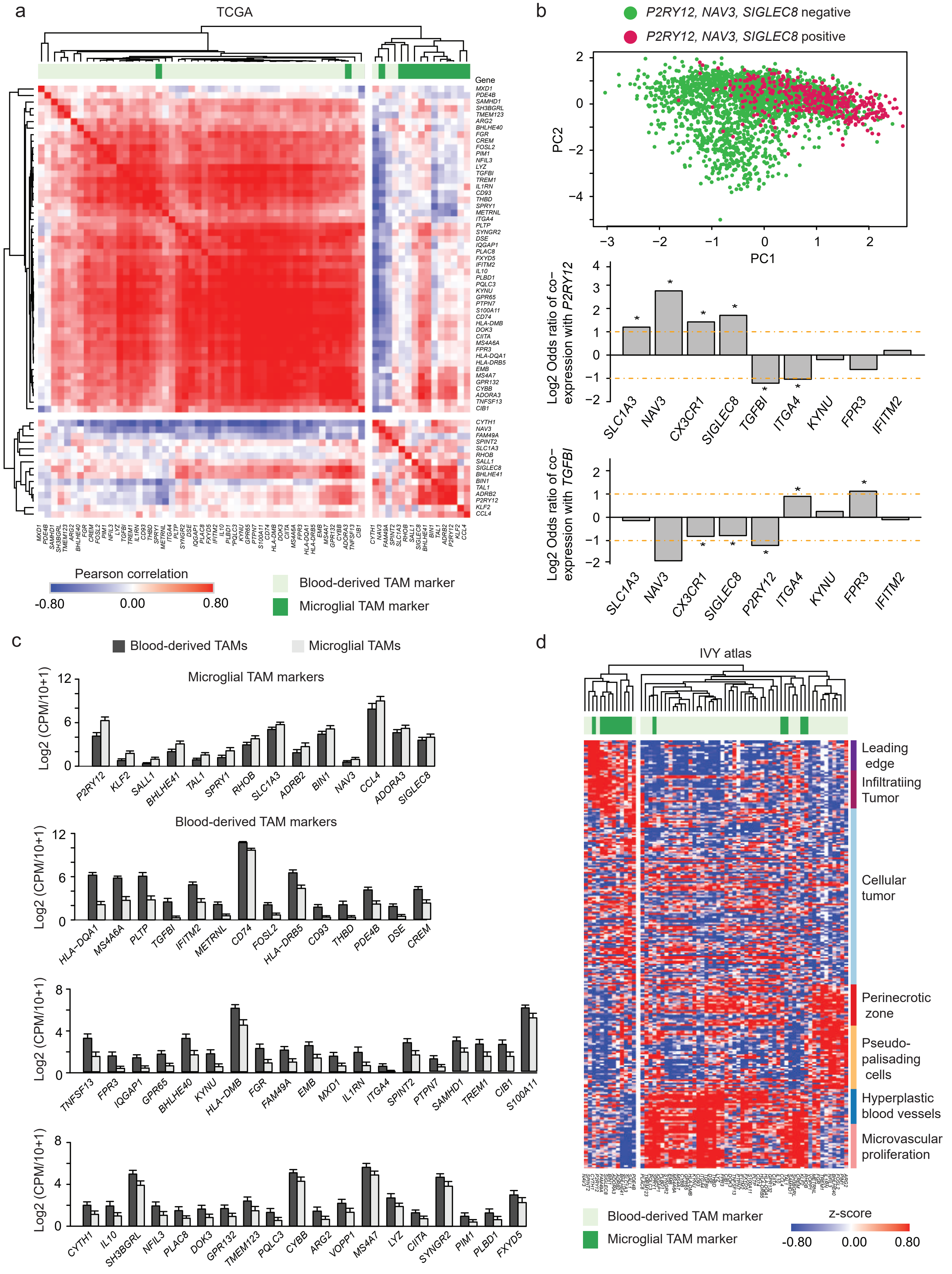
a
TCGA
Gene
Pearson correlation
-0.80
0.00
0.80
Blood-derived TAM marker
Microglial TAM marker
b
P2RY12, NAV3, SIGLEC8 negative
P2RY12, NAV3, SIGLEC8 positive
PC2
PC1
Log2 Odds ratio of co-expression with P2RY12
SLC1A3
NAV3
CX3CR1
SIGLEC8
TGFBI
ITGA4
KYNU
FPR3
IFITM2
Log2 Odds ratio of co-expression with TGFBI
SLC1A3
NAV3
CX3CR1
SIGLEC8
P2RY12
ITGA4
KYNU
FPR3
IFITM2
c
Blood-derived TAMs
Microglial TAMs
Microglial TAM markers
Log2 (CPM/10+1)
P2RY12
KLF2
SALL1
BHLHE41
TAL1
SPRY1
RHOB
SLC1A3
ADRB2
BIN1
NAV3
CCL4
ADORA3
SIGLEC8
Blood-derived TAM markers
HLA-DQA1
MS4A6A
PLTP
TGFBI
IFITM2
METRNL
CD74
FOSL2
HLA-DRB5
CD93
THBD
PDE4B
DSE
CREM
TNFSF13
FPR3
IQGAP1
GPR65
BHLHE40
KYNU
HLA-DMB
FGR
FAM49A
EMB
MXD1
IL1RN
ITGA4
SPINT2
PTPN7
SAMHD1
TREM1
CIB1
S100A11
CYTH1
IL10
SH3BGRL
NFIL3
PLAC8
DOK3
GPR132
TMEM123
PQLC3
CYBB
ARG2
VOPP1
MS4A7
LYZ
CIITA
SYNGR2
PIM1
PLBD1
FXYD5
d
IVY atlas
Leading edge
Infiltratiing Tumor
Cellular tumor
Perinecrotic zone
Pseudo-palisading cells
Hyperplastic blood vessels
Microvascular proliferation
Blood-derived TAM marker
Microglial TAM marker
z-score
-0.80
0.00
0.80

Figure S3

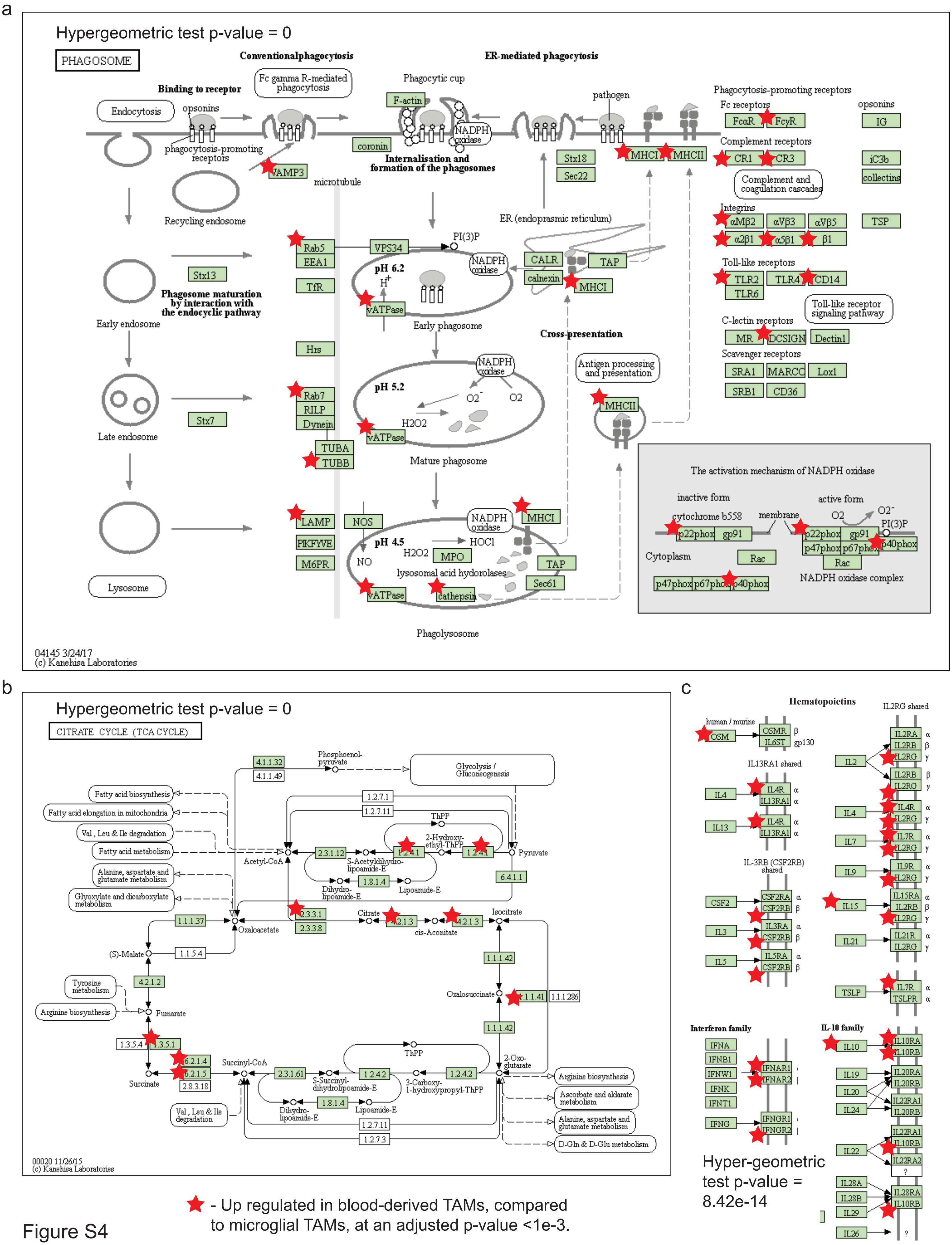

- Up regulated in blood-derived TAMs, compared to microglial TAMs, at an adjusted p-value <1e-3.

Figure S4

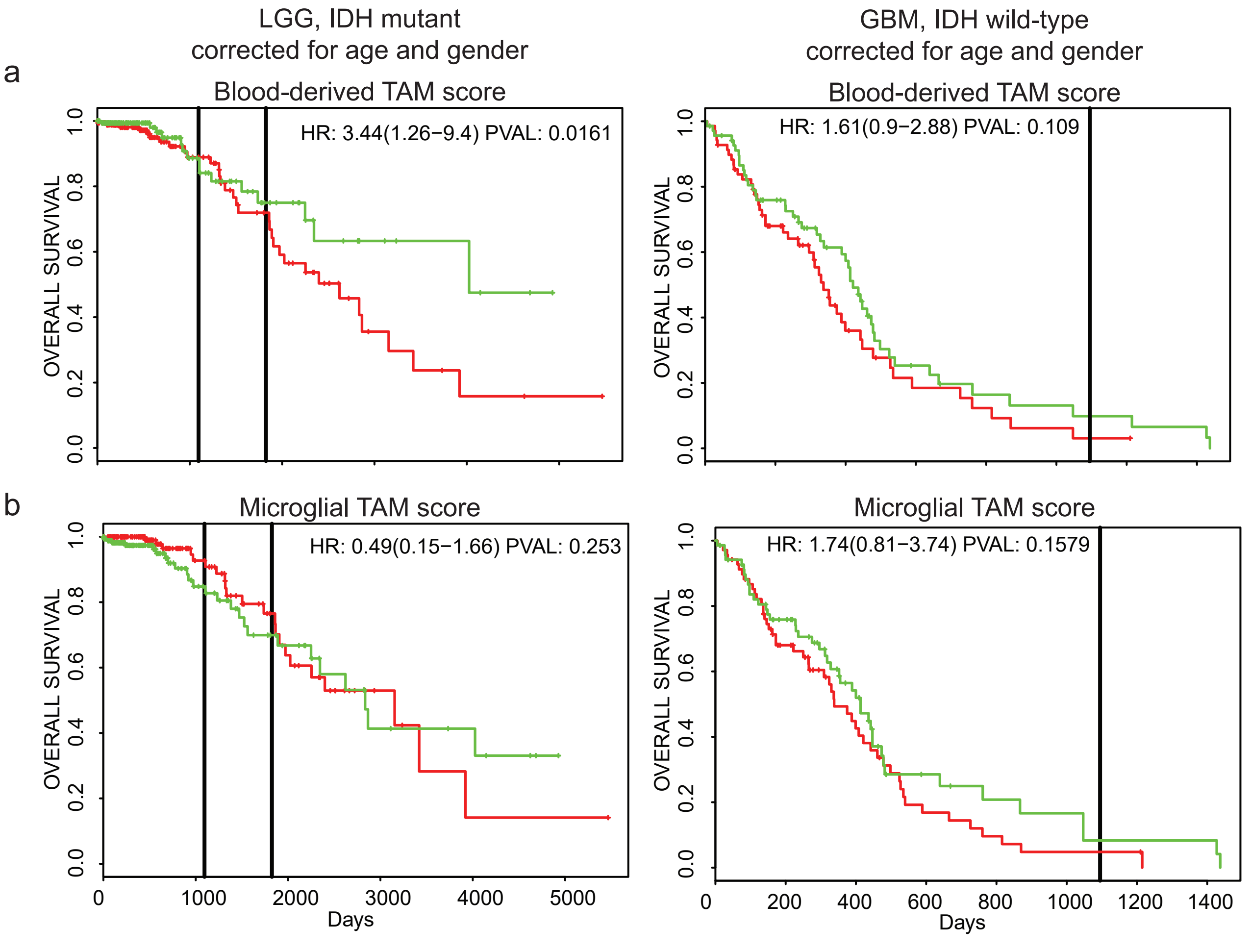
LGG, IDH mutant
corrected for age and gender
GBM, IDH wild-type
corrected for age and gender
a
Blood-derived TAM score
HR: 3.44(1.26−9.4) PVAL: 0.0161
HR: 1.61(0.9−2.88) PVAL: 0.109
b
Microglial TAM score
HR: 0.49(0.15−1.66) PVAL: 0.253
HR: 1.74(0.81−3.74) PVAL: 0.1579
OVERALL SURVIVAL
0.0
0.2
0.4
0.6
0.8
1.0
0
1000
2000
3000
4000
5000
200
400
600
800
1000
1200
1400
Days
High signature expression
Low signature expression

Figure S5

Figure S6

a

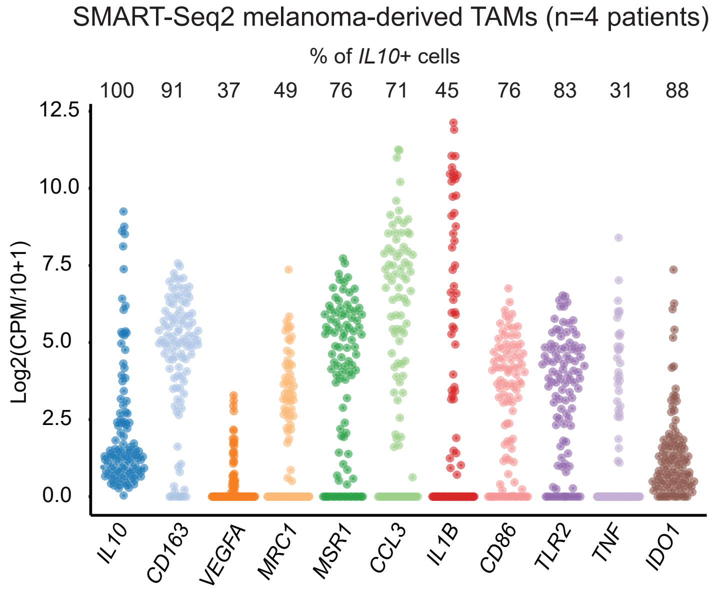

SMART-Seq2 melanoma-derived TAMs (n=4 patients)
% of IL10+ cells
100 91 37 49 76 71 45 76 83 31 88
Log2(CPM/10+1)
12.5
10.0
7.5
5.0
2.5
0.0
IL10
CD163
VEGFA
MRC1
MSR1
CCL3
IL1B
CD86
TLR2
TNF
IDO1

b

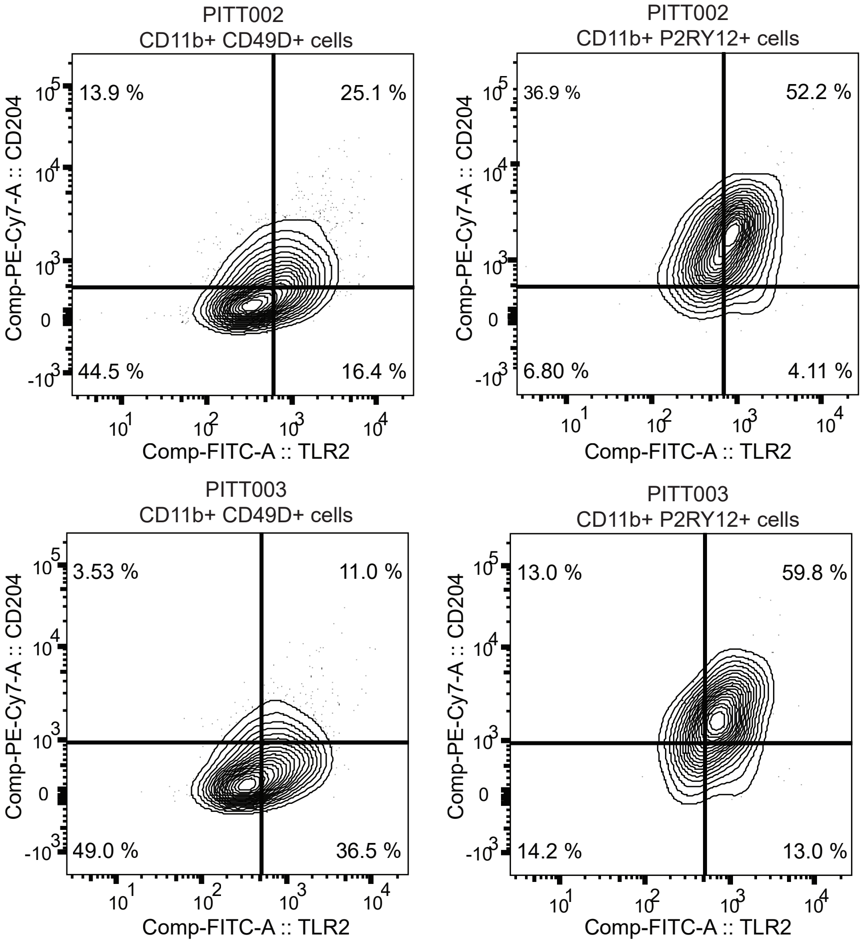

PITT002
CD11b+ CD49D+ cells
13.9 %
25.1 %
44.5 %
16.4 %
PITT002
CD11b+ P2RY12+ cells
36.9 %
52.2 %
6.80 %
4.11 %
PITT003
CD11b+ CD49D+ cells
3.53 %
11.0 %
49.0 %
36.5 %
PITT003
CD11b+ P2RY12+ cells
13.0 %
59.8 %
14.2 %
13.0 %
Comp-PE-Cy7-A :: CD204
Comp-FITC-A :: TLR2
